# Integrated Biomarker Assessment for Prognosis in Canine Mammary Carcinomas: Complementary Roles of Serum CA 15-3 and Immunohistochemistry Ki-67, and COX-2

**DOI:** 10.3390/ani16142208

**Published:** 2026-07-16

**Authors:** Breno Queiroz Pinheiro, Marcely Braga de Albuquerque, Francisco Emanuel Pinheiro Cavalcante, Isabela Reis Barroso do Nascimento, Fernanda Rezende Souza, Augusto Manuel Rodrigues Faustino, Lúcia Daniel Machado da Silva

**Affiliations:** 1 Laboratory of Carnivore Reproduction, Faculty of Veterinary Medicine, State University of Ceará (UECE), Fortaleza 60714-903, CE, Brazil; breno.queiroz@uece.br (B.Q.P.); marcely.albuquerque@aluno.uece.br (M.B.d.A.); 2 Residency Program in Small Animal Internal Medicine, Federal Rural University of the Semi-Arid Region (UFERSA), Mossoró 59625-900, RN, Brazil; emanuelp.cavalcante@aluno.uece.br; 3 Residency Program in Veterinary Clinical Pathology, São Paulo State University (UNESP), Botucatu 18618-682, SP, Brazil; isabela.reis@aluno.uece.br; 4 Department of Pathology, Institute of Biomedical Sciences, Federal University of Alfenas (UNIFAL), Alfenas 37130-001, MG, Brazil; fersouza.vet@gmail.com; 5 Department of Pathology and Molecular Immunology, Institute of Biomedical Sciences Abel Salazar (ICBAS), University of Porto, 4050-313 Porto, Portugal

**Keywords:** disease progression, immunohistochemistry, longitudinal monitoring, prognostic biomarker, risk stratification, serum biomarker

## Abstract

CMTs are biologically heterogeneous neoplasms for which reliable prognostic biomarkers remain limited. This study investigated the clinical significance of serum cancer antigen 15-3 (CA 15-3) and the immunohistochemical expression of Ki-67 and cyclooxygenase-2 (COX-2) in CMTs. Fifty-four female dogs with histologically confirmed CMTs and twelve healthy controls were prospectively evaluated. Serum CA 15-3 concentrations were measured before surgery and at 21 and 90 days post-mastectomy, while Ki-67 and COX-2 expression were assessed in tumor tissues. CA 15-3 was undetectable in controls and significantly higher in malignant than benign neoplasms (*p* < 0.05), with moderate correlations with clinical stage (s = 0.59), histological grade (s = 0.64), and the number of nodules (s = 0.42). Levels remained elevated in advanced stages despite surgery. ROC analysis identified a CA 15-3 cutoff of 3.01 IU/mL (AUC = 0.92) for discriminating malignant from benign tumors. Ki-67 correlated with histological grade (ρ = 0.41) but showed limited prognostic accuracy (AUC = 0.63). COX-2 expression lacked significant associations and showed poor discriminatory power (AUC = 0.44). Survival analyses did not identify significant differences among groups. Principal component analysis demonstrated clustering of aggressive CMTs according to biomarker profile. These findings suggest that serum CA 15-3 may represent a useful adjunct prognostic biomarker in canine mammary oncology, while the combination of serum and tissue biomarkers may improve prognostic stratification and may guide therapeutic decision-making in veterinary oncology. These findings support the growing role of biomarker panels in the clinical management of CMTs.

## Simple Summary

Canine mammary tumors (CMTs) are among the most common cancers in female dogs and remain a major cause of illness and death worldwide. One of the greatest challenges in clinical practice is identifying which tumors are likely to behave aggressively and require closer monitoring after surgery. Blood and tissue biomarkers may help veterinarians estimate tumor behavior beyond conventional histopathological assessment. In this study, we investigated whether a circulating biomarker and two tumor-associated proteins could provide additional information about disease severity and outcome. The results indicate that biomarker assessment may contribute to identifying dogs with more aggressive disease and support clinical decision-making during postoperative follow-up. Because canine mammary tumors share important biological similarities with human breast cancer, these findings may also contribute to the development and validation of comparative oncology approaches.

## 1. Introduction

Canine mammary tumors (CMTs) are among the most frequently diagnosed neoplasms in intact female dogs and represent a major challenge in veterinary oncology because of their high prevalence and marked biological heterogeneity [1]. Approximately half of CMTs are malignant, although this proportion may exceed 80% in some populations, and affected dogs can exhibit markedly different clinical outcomes despite presenting similar clinicopathological features [2,3]. Histological classification, grading, and TNM staging remain the cornerstones of prognostic assessment; however, considerable variability in disease progression, recurrence, and survival persists, highlighting the need for complementary prognostic tools [4,5,6].

Beyond their veterinary relevance, canine mammary carcinomas are increasingly recognized as a valuable spontaneous model for human breast cancer. Both diseases share epidemiological, histopathological, molecular, and clinical characteristics, making canine patients an important resource for comparative oncology and translational research [3,7]. Consequently, considerable efforts have been directed toward identifying biomarkers capable of improving prognostic stratification, monitoring disease progression, and supporting therapeutic decision-making [8,9,10].

Among the biomarkers investigated in mammary oncology, cancer antigen 15-3 (CA 15-3), a circulating mucin-type glycoprotein primarily derived from the MUC1 gene product, has emerged as one of the most promising circulating biomarkers. Widely used in the management of human breast cancer [11]. CA 15-3 has demonstrated potential utility in dogs as a minimally invasive indicator of tumor burden, disease aggressiveness, and clinical progression [12]. Previous studies have reported associations between elevated serum CA 15-3 concentrations and advanced clinical stage, increased tumor size, lymph node involvement, and other adverse prognostic parameters in CMTs [13,14,15]. However, despite encouraging results, its clinical application in veterinary medicine remains incompletely characterized, particularly with respect to longitudinal postoperative monitoring and long-term prognostic assessment [9].

In addition to circulating biomarkers, immunohistochemical markers have been extensively investigated as prognostic indicators in CMTs. Ki-67 is a nuclear protein expressed during the active phases of the cell cycle (G1, S, G2, and M) and is widely recognized as a marker of cellular proliferation [16]. An increased Ki-67 proliferative index has consistently been associated with higher histological grades, greater tumor aggressiveness, metastatic potential, and reduced survival, supporting its use as an indicator of biological behavior in mammary carcinomas [17,18,19,20]. Furthermore, physiological studies have demonstrated that Ki-67 expression varies according to the physiological dynamics of the mammary gland, reinforcing its biological relevance in both normal and neoplastic mammary tissues [21].

Cyclooxygenase-2 (COX-2) is an inducible enzyme involved in prostaglandin synthesis and tumor-associated inflammatory pathways [22]. Increased COX-2 expression has been linked to key hallmarks of cancer, including angiogenesis, cellular proliferation, immune modulation, tumor progression, and adverse clinical outcomes [23]. Moreover, the potential therapeutic benefit of COX-2 inhibition has stimulated interest in its use not only as a prognostic biomarker but also as a therapeutic target [24,25]. Previous studies have linked COX-2 overexpression to biological processes associated with tumor progression, including angiogenesis, cellular proliferation, immune modulation, invasion, and adverse clinical outcomes in canine mammary carcinomas [26,27,28,29,30].

Although CA 15-3, Ki-67, and COX-2 have individually demonstrated prognostic value, most previous studies have evaluated these biomarkers independently. Consequently, limited information is available regarding their integrated behavior within the same patient population and their combined relationship with clinicopathological characteristics and survival outcomes. Furthermore, studies simultaneously evaluating serum and tissue biomarkers with longitudinal postoperative follow-up remain scarce in canine mammary oncology [8,9,10].

Although histological grading and TNM staging remain essential tools for prognostic assessment in CMTs, these methods present inherent limitations, including inter-observer variability and their inability to fully capture the biological heterogeneity of these neoplasms. The incorporation of molecular biomarkers may refine prognostic stratification by providing complementary information on tumor proliferation, the inflammatory microenvironment, and systemic tumor burden.

In this context, the simultaneous evaluation of a circulating biomarker (CA 15-3) and tissue-based markers (Ki-67 and COX-2) offers a more comprehensive approach to characterizing tumor behavior and guiding clinical decision-making. We hypothesized that the integration of circulating and tissue biomarkers would provide complementary information for assessing tumor aggressiveness and prognostic stratification. Therefore, this study aimed to evaluate the prognostic value of integrating serum CA 15-3 levels with the immunohistochemical (IHC) expression of Ki-67 and COX-2 in canine mammary carcinomas, investigating their associations with clinicopathological variables and their ability to predict tumor aggressiveness and clinical outcomes.

## 2. Materials and Methods

This study was approved by the Animal Experimentation Ethics Committee of the State University of Ceará (UECE) (protocol no. 11690648/2021). Written informed consent was obtained from all owners prior to enrollment. The data supporting the findings of this study are available from the corresponding author upon reasonable request. No cell lines were used in this study.

### 2.1. Study Design

A total of 84 female dogs with CMTs were initially screened prospectively for inclusion in this study between June 2022 and August 2023 at the Veterinary Hospital Sylvio Barbosa Cardoso, State University of Ceará, Fortaleza, Brazil. Dogs with mammary nodules were initially screened by fine-needle aspiration cytology. Cases with cytological findings suggestive of mammary carcinoma were referred for surgical treatment and subsequent histopathological confirmation. Only dogs with histologically confirmed mammary carcinomas were included in the study. Other mammary neoplasms, including non-carcinomatous CMTs, were excluded. Additional inclusion criteria included the absence of previous chemotherapy, the availability of adequate tumor samples for histopathological and immunohistochemical analyses, and completion of the minimum follow-up evaluations established by the study protocol. Cases with incomplete follow-up, insufficient tissue samples, or previous oncological treatment were excluded.

The control group, consisting of healthy female dogs under controlled conditions, was established to provide a baseline for CA 15-3 levels in animals free of mammary neoplasia or systemic inflammatory processes. While this group ensures internal consistency for baseline measurements, we acknowledge the differences in age and breed composition compared with the CMT group, which were dictated by the availability of a healthy population with complete clinical records.

### 2.2. Clinical, Surgical, and Histopathological Evaluation

Each dog underwent a complete clinical examination, including lymph node palpation, tumor measurement, and assessment for ulceration or necrosis. Blood samples were collected for hematological and biochemical analysis. Thoracic radiography (three projections) and total abdominal ultrasound were performed to evaluate metastatic spread. A variety of mastectomy techniques were selected according to tumor size, fixation to surrounding tissues, and the number and distribution of lesions. Regional lymphadenectomy was routinely performed, with the corresponding inguinal and/or axillary lymph node(s) surgically excised together with the mammary specimen and submitted for histopathological analysis [31]. Histopathological evaluation was performed using hematoxylin and eosin (H&E) staining, including assessment of the tumor, regional lymph nodes, and surgical margins, the latter previously marked with India ink [4]. Histological grading was determined based on tubular formation, nuclear pleomorphism, and mitotic count [5].

All serum samples for CA 15-3 measurement and formalin-fixed, paraffin-embedded tumor tissue samples for immunohistochemical analysis were obtained from the same animals. Blood was collected at the time of anesthetic induction for mastectomy (D0), prior to tumor removal, and the tumor tissue used for IHC was obtained from the same surgical procedure. Therefore, the reported correlations between serum CA 15-3 levels and IHC markers (Ki-67 and COX-2) were derived from paired samples collected simultaneously from each individual patient.

### 2.3. Patient Follow-Up

All CMT cases included in this study underwent a structured follow-up protocol to assess clinical outcomes and survival. The total follow-up period extended from the day of mastectomy (D0) to the last recorded contact, with a maximum follow-up of 30 months. Three clinical and laboratory evaluations were performed at predefined time points, with laboratory reassessments conducted on the day of mastectomy (D0), at 21 days post-surgery (D1), and at three months post-surgery (D2). These evaluations included physical examinations, hematological and biochemical analyses (renal and hepatic evaluation), and measurement of CA 15-3 levels. Beyond the three-month postoperative period, the follow-up for up to 30 months was conducted through telephone interviews with owners, focusing on clinical status, potential tumor recurrence, and survival status. For all patients, the date of the last contact was recorded. In cases of confirmed death, both the date and the presumed cause of death were documented.

Overall survival was defined as the interval between mastectomy and death from any cause or the last recorded contact. Recurrence was defined as the reappearance of the tumor during postoperative monitoring at the surgical site and was confirmed by cytological evaluation.

### 2.4. Serum Biomarkers CA 15-3

Peripheral venous blood samples were obtained from all female dogs with CMTs at the time of anesthetic induction for mastectomy (D0), while the tumor was still present, and during routine checkups (D1 and D2), as well as on the day of the clinical evaluation for the control group. After collection and clot retraction, the samples were centrifuged at 1450× *g* for 5 min. Serum CA 15-3 measurements were performed according to the manufacturer’s protocol. All assays were performed in duplicate, and positive and negative controls (supplied with the kit) with known concentration ranges were included. CA 15-3 (IU/mL) levels were measured using a commercial kit (BR-MA, Siemens^®^, Erlangen, Germany) based on a chemiluminescent immunometric assay performed on the IMMULITE 1000 Immunoassay System (Siemens^®^) with a measurement range of 2 to 400 IU/mL. At the time of the study, no reference intervals had been established for serum CA 15-3 concentrations in healthy female dogs.

### 2.5. Immunohistochemistry (IHC) Analysis

In cases presenting multiple mammary carcinomas, the tumor selected for immunohistochemical evaluation corresponded to the lesion with the worst prognostic profile, based on histological grade, histological subtype, and established prognostic criteria described for canine mammary carcinomas [5,32,33].

IHC analysis was performed on 3 µm-thick sections obtained from formalin-fixed, paraffin-embedded CMT samples. For antigen retrieval, slides were incubated in 0.01 M sodium citrate buffer (pH 6.0) at 99.8 °C for 10 min, using a laboratory-grade slide steamer. Endogenous peroxidase activity was blocked using Peroxidase Block (Novolink Max Polymer Detection System, Leica Biosystems^®^, Wetzlar, Germany). A protein-blocking step was subsequently performed using Protein Block (Novolink™, Leica Biosystems^®^) to minimize non-specific antibody binding. The sections were incubated overnight at 4 °C with either a monoclonal mouse anti-Ki-67 antibody (clone MIB-1, Dako^®^, Glostrup, Denmark; M7240; 1:50) or a rabbit monoclonal anti-COX-2 antibody (clone SP21, Thermo Fisher Scientific^®^, Waltham, MA, USA; 1:100).

Detection was carried out using a polymer-based secondary antibody system (Novolink Max Polymer Detection System, Leica Biosystems^®^), followed by visualization with 3,3′-diaminobenzidine (DAB) chromogen and counterstaining with Mayer’s hematoxylin.

Appropriate positive and negative controls were included in all runs: known Ki-67- and COX-2-expressing canine tissues were used as positive controls, while negative controls were obtained by omission of the primary antibody.

Ki-67 proliferative index was determined by manual counting of 1000 cells in hotspot areas under 400× magnification, following the conventional methodology adopted in previous canine mammary tumor studies [16]. The proliferative index was expressed as the percentage of positively stained nuclei [18].

COX-2 expression was evaluated semi-quantitatively, considering both the proportion of stained cells and the intensity of cytoplasmic staining in five high-power fields (400×). Distribution scores were defined as follows: 0 (absent), 1 (≤10%), 2 (11–30%), 3 (31–60%), and 4 (>60%). Staining intensity was graded from 0 (negative) to 3 (strong). The final score, obtained by multiplying distribution and intensity scores (range 0–12), was categorized as low (0–5) or high (6–12) [17]. The immunohistochemical scoring system for Ki-67 and COX-2 is summarized in Table 1.

All immunohistochemical evaluations were performed by a single observer who was blinded to clinical and pathological data at the time of scoring to minimize potential bias.

### 2.6. Statistical Analysis

Statistical analyses were performed using R (v.4.3.0) and Python (v.3.9), with *p* < 0.05 considered statistically significant. Data distribution and variance homogeneity were evaluated using the Shapiro–Wilk, Kolmogorov–Smirnov, and Levene’s tests, and appropriate statistical methods were applied accordingly. Differences in CA 15-3 levels (D0, D1, and D2), Ki-67, and COX-2 expression between clinical stages were assessed using the Kruskal–Wallis test, with Bonferroni correction for multiple comparisons. Longitudinal variations in CA 15-3 levels were analyzed using the Friedman test, and Spearman’s correlation was used to evaluate associations with CMT-related variables. Kaplan–Meier survival curves were generated for clinical staging, with group comparisons performed using the log-rank test. Additionally, receiver operating characteristic (ROC) curves were used to determine the optimal cutoff values for CA 15-3, Ki-67, and COX-2 for distinguishing malignant from benign CMTs, while principal component analysis (PCA) was applied to explore clustering patterns.

### 2.7. GenAI Statement

During the preparation of this manuscript, the authors used ChatGPT (OpenAI, GPT-5 series) to assist with language refinement, text organization, manuscript formatting, and editorial adaptation to the journal guidelines. The authors reviewed and edited all generated content and take full responsibility for the accuracy, interpretation, and integrity of the final manuscript. No generative artificial intelligence tools were used for study design, data collection, data analysis, data interpretation, or drawing scientific conclusions.

## 3. Results

### 3.1. Animal Selection

The selected dogs were divided into six groups, including a control group consisting of twelve clinically healthy Australian Shepherd female dogs from a commercial breeding kennel. This population was selected to minimize biological variability, ensure homogeneous management conditions, and provide access to complete clinical records. All control dogs underwent clinical and laboratory evaluations to confirm the absence of CMTs and other relevant systemic diseases. Due to the absence of confirmatory tests establishing that the nodules observed on imaging were metastases from CMTs, patients with stage V disease were excluded. The remaining CMT groups were subdivided as follows:

- Benign (B): Dogs with histopathologically confirmed only benign mammary epithelial lesions (9/54).
- Stage I: Carcinomas measuring ≤3 cm in diameter, without lymph node or distant metastasis (11/54).
- Stage II: Carcinomas measuring >3 cm and ≤5 cm in diameter, without lymph node or distant metastasis (13/54).
- Stage III: Carcinomas measuring >5 cm in diameter, without lymph node or distant metastasis (11/54).
- Stage IV: Carcinomas of any size with confirmed regional lymph node metastases (10/54).

The subdivision followed the TNM system and the guidelines of the World Health Organization (WHO) [6].

### 3.2. Population Characteristics

The control group consisted of intact female dogs with a mean age of 2.33 ± 0.65 years and a mean body weight of 18.83 ± 3.89 kg. Among the 54 CMT cases, the mean age was 9.85 ± 3.43 years, and the mean body weight was 10.12 ± 7.35 kg. Mixed-breed dogs were the most common breed (51.85%), followed by Pinscher (14.81%), Poodle (9.26%), and Yorkshire Terrier (5.56%). Most dogs were intact (83.33%). Of the 54 CMT cases, 16.67% were classified as benign and 83.33% as malignant tumors. Stage II was the most frequent clinical stage, followed by stages I, III, and IV (Table 1). Histological grade differed significantly among clinical stages (*p* = 0.01), with higher grades observed in more advanced stages. Post hoc analysis identified significant differences between stages I and IV (*p* = 0.01) and between stages II and IV (*p* = 0.04) (Table 2).

### 3.3. Serum CA 15-3 According to Groups

Serum CA 15-3 levels in the control group were below the detection threshold of the assay, preventing quantitative measurement. This result is consistent with our previous findings, where CA 15-3 concentrations in healthy dogs were either undetectable or at the lower limit of detection [12]. For statistical analyses, values below the detection limit were assigned the lowest detectable value of the assay. Accordingly, statistically significant differences were observed between the control group and all other groups.

Serum CA 15-3 levels at D0 differed significantly among clinical stages (*p* < 0.01), with higher concentrations observed in more advanced stages (Table 2). CA 15-3 levels were significantly higher in stages II, III, and IV than in the benign group (*p* < 0.01 for all comparisons), whereas no significant difference was observed between the benign group and stage I (*p* > 0.05). Additionally, stage IV showed significantly higher CA 15-3 levels than stage II (*p* < 0.01), while no differences were detected among stages I–III (*p* > 0.05) (Table 2).

Similar patterns were observed at D1 (21 days post-mastectomy) and D2 (3 months post-surgery), with significant differences across clinical stages (*p* < 0.01 for both time points). In both evaluations, CA 15-3 levels remained significantly higher in stages II, III, and IV compared to benign tumors (*p* < 0.01), whereas stage I did not differ from the benign group (*p* > 0.05). Similarly, stage IV differed significantly from stage II (*p* < 0.01), with no significant differences among the remaining stages (*p* > 0.05), indicating a consistent association between increasing CA 15-3 levels and tumor progression over time (Table 3).

### 3.4. Temporal Changes in CA 15-3 Concentrations

Longitudinal analysis of CA 15-3 levels over time (D0 to D1 to D2) revealed significant changes within all clinical groups, including benign tumors and stages I–IV (*p* < 0.01 for all groups). Post hoc pairwise comparisons demonstrated a significant reduction in CA 15-3 levels between all time points across all groups (*p* < 0.05 for all comparisons) (Table 2).

### 3.5. Immunohistochemical Expression of Ki-67 and COX-2

**Figure 1:**
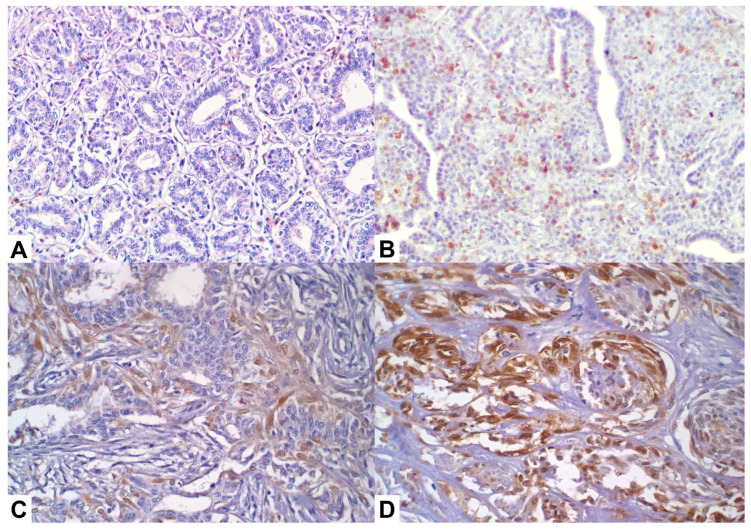
Representative microphotographs of immunohistochemical expression of Ki-67 and COX-2 observed in the study. (**A**) Fibroadenoma with low Ki-67 expression (20× objective); (**B**) Ductal carcinoma with high Ki-67 expression (20× objective); (**C**) Micropapillary carcinoma with low COX-2 expression (40× objective); (**D**) Carcinoma in mixed tumor with high COX-2 expression (40× objective); all detections were carried out with DAB chromogen, followed by counterstaining with Mayer’s hematoxylin, with positive immunoreactivity indicated by brown staining.

No significant differences in Ki-67 or COX-2 immunohistochemical expression were observed among clinical groups (*p* > 0.05 for all comparisons) (Figure 1).

### 3.6. Correlations Between Biomarkers and Clinicopathological Variables

Spearman’s correlation analysis demonstrated that CA 15-3 (D0) was moderately correlated with CMT stage (s = 0.59, *p* < 0.0001), histological grade (s = 0.64, *p* < 0.0001), and the number of nodules (s = 0.42, *p* = 0.001). However, no correlation was found between CA 15-3 and Ki-67 (s = 0.13, *p* > 0.05). Similarly, necrosis and ulceration did not show significant correlations with CA 15-3 levels. Ki-67 was significantly correlated only with CMTs grade (s = 0.41, *p* = 0.005), but not with clinical stage, tumor size, or CA 15-3 levels (*p* > 0.05 for all comparisons). COX-2 expression did not show significant correlations with any of the analyzed variables (*p* > 0.05 for all comparisons).

### 3.7. Diagnostic Performance of CA 15-3, Ki-67, and COX-2

**Figure 2:**
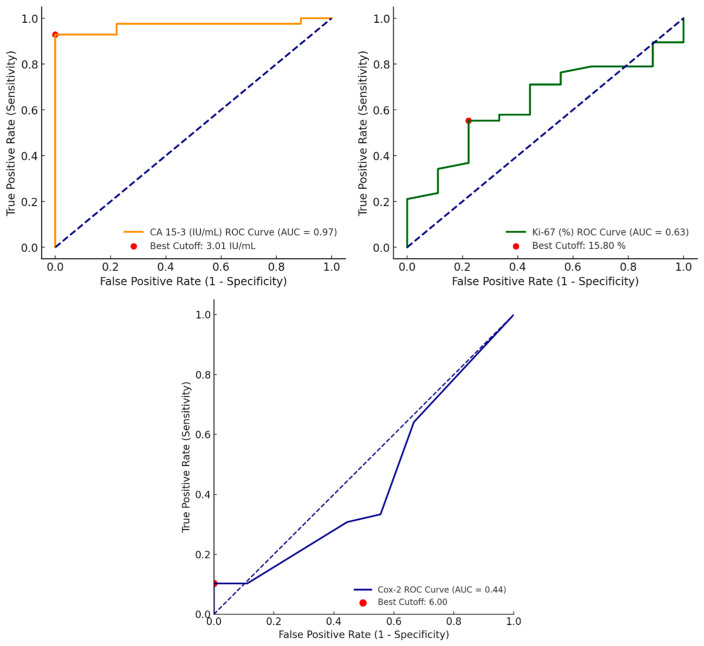
Receiver operating characteristic (ROC) analysis for CA 15-3, Ki-67, and COX-2 in distinguishing malignant from benign CMTs. The upper left panel (yellow) displays the ROC curve for CA 15-3; the upper right panel (green) shows the ROC curve for Ki-67; and the lower panel (blue) presents the ROC curve for COX-2. The dashed diagonal line represents the chance level of discrimination (AUC = 0.5).

Receiver operating characteristic (ROC) analysis identified an optimal CA 15-3 cutoff value of 3.01 IU/mL, with high sensitivity (93.0%) and specificity (90.9%) for distinguishing malignant from benign CMTs (AUC = 0.92). Ki-67 showed moderate discriminatory performance, with an optimal cutoff of 15.8%, sensitivity of 56.1%, and specificity of 77.8% (AUC = 0.63). The ROC curve for COX-2 demonstrated poor discriminatory performance with an optimal cutoff of an IHC score of 6.00, yielding 66.7% a sensitivity and 33.3% a specificity (AUC = 0.44) (Figure 2).

### 3.8. Survival Analysis

**Figure 3:**
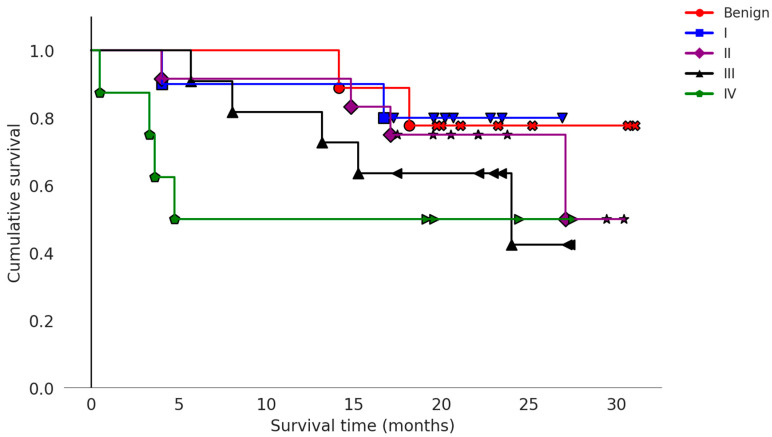
Kaplan–Meier survival analysis of CMTs across stage groups. This Kaplan–Meier survival curve illustrates the cumulative survival probability over time (in months) for CMTs stratified by clinical staging (benign, I, II, III, and IV). Each line represents a different staging group, with distinct colors and markers differentiating them.

Kaplan–Meier survival analysis stratified among clinical stages revealed no significant differences among groups (*p* = 0.56) (Figure 3).

### 3.9. Principal Component Analysis

PCA incorporating histological grade, number of nodules, ulceration, necrosis, diameter, clinical stage, CA 15-3 levels, Ki-67, and COX-2 scores revealed that the first two principal components explained 58.26% of the total variance. PC1 accounted for 40.30% of the variance and was predominantly influenced by clinical staging, histological grade, CA 15-3 levels, number of nodules, and diameter. PC2 explained 17.96% of the variance and was mainly associated with Ki-67, COX-2, ulceration, and necrosis.

**Figure 4:**
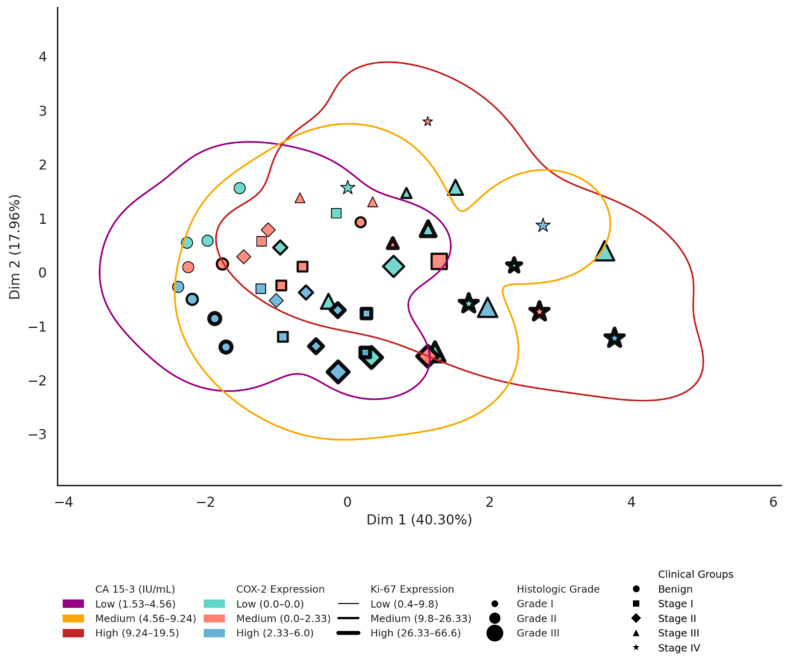
Multidimensional distribution of clinical and pathological parameters across CA 15-3 terciles. CMTs are grouped according to serum CA 15-3 concentration terciles, represented by colored contours: low (≤4.56), medium (4.56–9.24), and high (≥9.24). Each point corresponds to a single patient and simultaneously encodes several dimensions of CMTs behavior: the color reflects COX-2 expression level (low to high), the thickness of the black outline represents Ki-67 proliferative index (thicker borders indicate higher proliferation), the size of the point corresponds to histologic grade (from grade I to III), and the shape indicates clinical classification (benign, or malignant stages I–IV).

A multivariable PCA biplot with biomarker expression tertiles, histological grade, and clinical staging demonstrated intersecting but distinguishable clusters. CMTs with higher CA 15-3 concentrations, Ki-67 proliferative index, and COX-2 scores were more clustered toward the right side of the PC1 axis, close to high histological grades and advanced clinical stages (Figure 4).

## 4. Discussion

### 4.1. Main Findings

The present study evaluated the prognostic relevance of serum CA 15-3 levels and the immunohistochemical expression of Ki-67 and COX-2 in canine mammary carcinomas based on clinicopathological, survival, and multivariate analyses. Overall, serum CA 15-3 demonstrated associations with advanced clinical stage, higher histological grade, and a greater number of mammary nodules. In contrast, the Ki-67 proliferative index was associated with histological grade, whereas COX-2 expression was associated with ulceration and necrosis.

These findings support the hypothesis that circulating and tissue biomarkers provide complementary information regarding tumor biology. This concept is aligned with current trends in canine mammary oncology, where integrated biomarker panels are increasingly proposed to improve prognostic stratification beyond conventional clinicopathological parameters [8,9,10].

### 4.2. Clinical Significance of CA 15-3

Among the evaluated biomarkers, serum CA 15-3 demonstrated the strongest association with clinicopathological indicators of tumor progression. Increased CA 15-3 concentrations were correlated with advanced clinical stage, higher histological grade, and a greater number of mammary nodules, suggesting that this biomarker reflects both tumor burden and biological aggressiveness. Similar associations have been reported in previous studies, where elevated CA 15-3 concentrations were linked to malignant behavior, tumor progression, and adverse prognostic characteristics in CMTs [13,14,15].

An additional finding of the present study was the absence of detectable CA 15-3 concentrations in clinically healthy dogs. Although reference intervals for canine CA 15-3 have not yet been fully established, our previous investigations have similarly reported very low or undetectable serum concentrations in healthy animals, reinforcing the potential utility of this marker for distinguishing physiological from neoplastic conditions [13].

The diagnostic performance observed in the ROC analysis further supports the clinical relevance of CA 15-3. The proposed cutoff value of 3.01 IU/mL yielded high sensitivity and specificity for differentiating malignant from benign tumors, resulting in an AUC of 0.92. These results are consistent with growing evidence suggesting that CA 15-3 may serve as a useful biomarker in the diagnostic and prognostic assessment of canine mammary carcinomas [8,9,10].

Interestingly, serum CA 15-3 concentrations decreased significantly following surgical treatment in all clinical groups evaluated. This reduction likely reflects decreased tumor burden after mastectomy and suggests that serial measurements may offer additional insights into disease progression and treatment response, warranting further investigation. Longitudinal evaluation of circulating biomarkers remains relatively unexplored in canine mammary oncology, and additional studies are required to determine whether persistent elevation or renewed increases in CA 15-3 concentrations may predict recurrence or disease progression.

Despite these promising findings, CA 15-3 did not demonstrate statistically significant associations with overall survival in the present cohort. Visual inspection of the Kaplan–Meier curves suggested lower survival probabilities among dogs with more advanced disease; however, these differences did not reach statistical significance. The lack of significant survival differences in the present study reinforces the need for a multimodal approach that integrates serum and tissue biomarkers for more precise prognostication.

### 4.3. Prognostic Role of Ki-67

Ki-67 proliferative index was positively associated with higher histological grades and increased aggressiveness, including necrosis and ulceration, reinforcing its role as a marker of cellular proliferation and tumor aggressiveness in CMTs. This finding is consistent with previous studies that identified increased Ki-67 expression in high-grade mammary carcinomas and demonstrated associations with unfavorable pathological characteristics and reduced survival [16,17,18,19,20].

The association between Ki-67 and histological grade observed in the present study, together with its lack of correlation with tumor size, clinical stage, and serum CA 15-3 concentrations, reflects the distinct biological processes. Ki-67 quantifies the fraction of actively proliferating tumor cells, whereas tumor size and clinical stage represent cumulative tumor growth and disease dissemination, and CA 15-3 primarily reflects tumor burden rather than proliferative activity. Consequently, these biomarkers provide complementary information. Similar heterogeneity in the prognostic performance of Ki-67 has been reported previously, with stronger and more consistent associations with histological grade than with clinical stage or tumor size, and variable diagnostic accuracy across studies [10,12,13].

An additional aspect that deserves consideration is the biological variability of Ki-67. Previous work from our research group demonstrated that proliferative activity in normal canine mammary tissue varies throughout the estrous cycle, indicating that Ki-67 expression may be influenced by physiological hormonal modulation [21]. Although most female dogs included in the present study were intact, information regarding the specific stage of the reproductive cycle was not available, preventing further evaluation of its potential influence on tumor proliferative indices.

Another important limitation of Ki-67 is its dependence on tissue sampling and immunohistochemical evaluation. Unlike circulating biomarkers, which can be repeatedly assessed during clinical follow-up, Ki-67 requires tumor tissue acquisition and may be affected by intratumoral heterogeneity and hotspot selection. These factors may contribute to variability among studies and partially explain why Ki-67 consistently correlates with histological grade but exhibits less consistent associations with survival outcomes and clinical staging.

The limited prognostic performance of Ki-67 in our cohort, as reflected by the moderate AUC of 0.63, warrants critical consideration. Several factors may explain why Ki-67, despite its established association with proliferation, showed only moderate discriminatory power in our study. First, Ki-67 expression is inherently variable within tumors due to intratumoral heterogeneity, and hotspot-based manual counting may not fully capture the global proliferative activity of the neoplasm. Second, the relatively small sample size and the predominance of intermediate-grade tumors may have reduced the dynamic range of Ki-67 values, limiting its ability to discriminate between prognostic groups. Third, Ki-67 reflects only one dimension of tumor biology, proliferative activity, whereas clinical outcomes in CMTs are influenced by multiple additional factors, including metastatic potential, the immune microenvironment, and molecular subtype.

The lack of correlation between Ki-67 and tumor size, clinical stage, and CA 15-3 levels deserves specific discussion. The absence of association with tumor size and clinical stage suggests that proliferative activity and anatomical tumor extension represent distinct biological dimensions. Whereas Ki-67 captures the intrinsic proliferative potential of neoplastic cells, the clinical stage reflects the cumulative effect of tumor growth, invasion, and dissemination over time. The lack of correlation with CA 15-3 is also biologically plausible, as CA 15-3 is a circulating marker of tumor burden and secretory activity, whereas Ki-67 is a tissue-based marker of cell cycle activity; these markers may reflect complementary rather than overlapping aspects of tumor biology. Similar patterns have been reported in human breast cancer, where Ki-67 consistently correlates with histological grade but shows variable associations with other clinicopathological parameters [34,35]. These observations reinforce the concept that Ki-67 should be interpreted within a multiparametric framework rather than as a standalone prognostic tool.

Taken together, the present findings support the use of Ki-67 as an indicator of tumor proliferative activity and histological aggressiveness. However, its greatest clinical value appears to emerge when interpreted alongside complementary clinicopathological and molecular markers rather than as an independent prognostic tool. The PCA results reinforce the complementary nature of these biomarkers in characterizing tumor biology.

### 4.4. COX-2 and Tumor-Associated Inflammation

Unlike CA 15-3 and Ki-67, COX-2 expression was not significantly associated with clinical stage, histological grade, tumor size, or survival outcomes in the present study. Furthermore, COX-2 demonstrated poor discriminatory performance for distinguishing malignant from benign tumors, as demonstrated by the low area under the ROC curve (AUC = 0.44). These findings suggest that COX-2 expression alone may have limited value as an independent prognostic biomarker in the evaluated cohort.

The absence of significant associations in the present study may reflect the biological and methodological heterogeneity commonly observed in CMT research. Differences in histological subtype composition, tumor grade distribution, sample size, scoring systems, antibody clones, and positivity thresholds may substantially influence reported results. In addition, previous studies have frequently reported associations between COX-2 overexpression and adverse clinicopathological characteristics in canine mammary carcinomas. Increased COX-2 expression has been linked to enhanced angiogenesis, tumor progression, inflammatory infiltrates, lymph node metastasis, and reduced survival, supporting its proposed role in mammary carcinogenesis and tumor aggressiveness [26,27,28,29,30].

An important methodological aspect of the present study is that none of the included patients received corticosteroids or non-steroidal anti-inflammatory drugs before surgery. Although this approach minimized potential pharmacological interference in COX-2 expression, intrinsic biological variability among tumors may still have influenced the observed results. Moreover, inflammatory signaling pathways are highly dynamic and may not be fully captured through a single immunohistochemical assessment performed at the time of surgical excision [23].

Despite the lack of significant univariable associations, principal component analysis revealed that COX-2 contributed predominantly to the second principal component, together with ulceration, necrosis, and the Ki-67 proliferative index. This finding suggests that COX-2 may reflect biological processes associated with tumor microenvironment interactions and inflammatory responses rather than directly mirroring tumor burden or clinical stage. Consequently, its biological relevance may become more evident when interpreted within a multiparametric framework rather than as an isolated biomarker. An important aspect of our findings is that the absence of statistically significant associations for COX-2, a negative result, does not diminish its biological relevance. On the contrary, the lack of correlation between COX-2 expression and clinical stage, histological grade, or survival in our cohort highlights a key point: individual biomarkers may perform differently depending on the specific patient population, tumor subtype composition, and methodological approaches. By transparently reporting these negative findings, we reinforce the interpretation that no single biomarker is sufficient for prognostic stratification in CMTs. The integration of multiple markers, each capturing different biological processes, tumor burden (CA 15-3), proliferation (Ki-67), and inflammation (COX-2), provides a more robust and clinically meaningful assessment than any individual marker alone.

The potential clinical importance of COX-2 should not be rejected solely based on its prognostic performance. Beyond its role as a biomarker, COX-2 remains a potential therapeutic target in canine mammary oncology. Experimental and clinical studies have demonstrated that selective COX-2 inhibition may interfere with tumor-associated inflammatory pathways and contribute to antitumor effects, supporting continued interest in this molecule as part of future therapeutic strategies [24,25].

Although COX-2 demonstrated poor prognostic discriminatory performance in our cohort (AUC = 0.44), its biological relevance should not be dismissed. It is important to distinguish between prognostic and predictive utility: while COX-2 expression alone was insufficient to stratify prognosis in this study, its overexpression in a subset of tumors supports continued investigation as a potential predictive marker for targeted therapy with selective COX-2 inhibitors (NSAIDs), as suggested by previous studies [24,25]. The therapeutic potential of COX-2 inhibition is based on its role in tumor-associated inflammatory pathways, angiogenesis, and immune modulation, mechanisms that are distinct from its utility as a prognostic discriminator. Therefore, these two applications, prognosis and therapeutic targeting, should be evaluated independently. Collectively, these findings indicate that COX-2 expression alone was insufficient to stratify prognostic risk in the present study. However, its contribution to multivariate analyses and its biological involvement in tumor-associated inflammation support further investigation of its role within integrated biomarker panels and targeted therapeutic approaches.

### 4.5. Integrative Biomarker Assessment

To the best of our knowledge, this is the first study to integrate serial serum CA 15-3 concentrations and the immunohistochemical expression of Ki-67 and COX-2 assessment in a single cohort of CMTs using clinicopathological, survival, and multivariate analyses. The present study demonstrates that these markers contribute distinct but complementary information regarding tumor biology and disease progression. The distribution of advanced-stage tumors within the PCA space also supports the notion that tumor aggressiveness is multifactorial and cannot be fully characterized by a single biomarker.

These observations are consistent with contemporary concepts in comparative and translational oncology, where biomarker panels are increasingly preferred over isolated markers for prognostic assessment and therapeutic decision-making [8,9,10]. Similar approaches have been adopted in human breast cancer, where combinations of circulating, histopathological, and molecular markers provide more accurate characterization of tumor behavior than individual variables alone [36].

The comparative nature of canine mammary carcinomas further enhances the relevance of these findings. Because canine and human mammary tumors share important epidemiological, pathological, and molecular characteristics, integrated biomarker approaches developed in veterinary medicine may contribute to comparative oncology research while simultaneously improving clinical management of canine patients [1,7].

### 4.6. Limitations and Perspectives

Some limitations should be considered when interpreting the findings of the present study. First, although the sample size was sufficient to identify significant associations between biomarkers and clinicopathological variables, the number of cases and the duration of follow-up may have limited the statistical power of survival analyses, potentially explaining the lack of significant differences in the Kaplan–Meier curves despite the biological aggressiveness observed in advanced stages. Second, as a single-center study, the external validity of our findings should be confirmed in larger, multicenter cohorts.

Regarding the diagnostic performance of CA 15-3, the proposed cutoff value of 3.01 IU/mL was derived and tested within the same cohort. Therefore, this cutoff should be considered preliminary and requires independent validation in geographically distinct populations. Furthermore, the reliance on manual hotspot-based scoring for Ki-67 and COX-2, while performed by a single blinded observer to ensure consistency, remains subject to intratumoral heterogeneity.

Future studies incorporating digital image analysis alongside standardized manual assessment, as well as longitudinal monitoring with longer follow-up periods, will be essential to fully establish the clinical utility of this integrated biomarker panel in canine mammary oncology.

## 5. Conclusions

This study demonstrates that serum CA 15-3 levels, the Ki-67 proliferative index, and COX-2 expression are relevant biomarkers associated with clinical and pathological features of CMTs. Elevated CA 15-3 concentrations, particularly on day 0 (D0), were significantly correlated with advanced clinical stages. Furthermore, the observed reduction in CA 15-3 concentrations following surgical treatment highlights the potential utility of longitudinal biomarker monitoring in clinical practice. Ki-67 expression was positively associated with histological grade, reinforcing its prognostic value. Despite COX-2 showing a weaker correlation with clinical features, its IHC scoring exhibited moderate alignment with CMT progression parameters in principal component analysis.

The combination of serum and tissue biomarkers may improve prognostic stratification and better guide therapeutic decision-making in veterinary oncology than either biomarker alone. These findings support the growing role of biomarker panels in the clinical management of CMTs and emphasize their potential translational relevance to human breast cancer.

## Figures and Tables

**Table 1:** Scoring criteria for Ki-67 and COX-2 IHC markers.

Marker	Scoring Method	Score/Categories	Description
Ki-67	Manual counting of 1000 cells in hotspot areas (400×)	Percentage (0–100%)	Proliferative index expressed as a percentage of positively stained nuclei
COX-2	Semi-quantitative: distribution (0–4) × intensity (0–3)	Final score 0–12; categorized as low (0–5) or high (6–12)	Distribution: 0 = absent, 1 = ≤10%, 2 = 11–30%, 3 = 31–60%, 4 = >60%. Intensity: 0 = negative, 1 = weak, 2 = moderate, 3 = strong

**Table 2:** Clinical and histopathological indices in female dogs with CMTs, according to study groups.

	B	I	II	III	IV
Most frequent breed	Pinscher (33.33%)	Mixed-breed (54.5%)	Mixed-breed (53.8%)	Mixed-breed (58.3%)	Mixed-breed (62.5%)
Age (years)	8.4 ± 3.9	11.5 ± 4.3	8.8 ± 3.0	9.3 ± 2.6	11.4 ± 2.3
Weight (kg)	9.4 ± 9.7	7.7 ± 4.1	9.2 ± 5.3	14.3 ± 10.5	9.4 ± 3.9
Reproductive status	Intact (100%)	Intact (63.6%)	Intact (100%)	Intact (83.3%)	Intact (75.0%)
Number of nodules	1.2 ± 0.4	2.3 ± 1.1	1.8 ± 1.3	2.3 ± 1.0	3.1 ± 2.3
Most affected mammary gland	M5 (55.5%)	M3 (36.3%)	M5 (38.5%)	M5 (41.7%)	M5 (50.0%)
Nodule diameter (cm)	1.4 ± 1.5	1.3 ± 0.7	2.7 ± 0.9	5.1 ± 3.2	7.5 ± 5.3
Most frequent diagnosis	Complex adenoma (33.33%)	Complex carcinoma (54.5%)	Mixed tumor carcinoma (38.4%)	Complex carcinoma (25.0%)	Comedocarcinoma (25.0%)
Grade	†	1.2 ± 0.4 ^A^	1.4 ± 0.5 ^A^	1.8 ± 0.9 ^B^	2.2 ± 0.7 ^B^

The table presents the distribution of clinical and histopathological variables in female dogs with CMTs, stratified into benign neoplasms (B) and carcinomas according to clinical staging (I, II, III, and IV). Inguinal mammary gland (M5) and cranial abdominal mammary gland (M3). Numerical values are presented as mean ± standard deviation, and relative frequencies are expressed as percentages (%). † Not applicable to this subgroup of the population. Superscript letters within the same row indicate statistically significant differences.

**Table 3:** Biomarker values for CA 15-3 at different time points (D0, D1, and D2), Ki-67, and COX-2 in female dogs with CMTs, according to study groups.

Staging	CA 15-3 (D0) (IU/mL)	CA 15-3 (D1) (IU/mL)	CA 15-3 (D2) (IU/mL)	Ki-67 (%)	COX-2 (Score)
B	2.32 ± 0.46 ^Aa^	1.99 ± 0.37 ^Ab^	1.62 ± 0.30 ^Ac^	12.43 ± 11.88 ^A^	1.78 ± 1.56 ^A^
I	7.71 ± 3.15 ^ABa^	6.70 ± 2.76 ^ABb^	5.41 ± 2.20 ^ABc^	14.93 ± 12.78 ^A^	2.11 ± 1.83 ^A^
II	5.80 ± 2.31 ^Ba^	5.16 ± 2.14 ^Bb^	4.23 ± 1.78 ^Bc^	24.38 ± 23.05 ^A^	2.18 ± 2.23 ^A^
III	9.71 ± 5.11 ^BCa^	8.38 ± 4.58 ^BCb^	6.58 ± 3.74 ^BCc^	21.84 ± 15.67 ^A^	0.60 ± 0.97 ^A^
IV	11.03 ± 5.16 ^Ca^	9.93 ± 4.79 ^Cb^	8.32 ± 4.30 ^Cc^	26.75 ± 19.52 ^A^	1.50 ± 2.14 ^A^

The table presents the mean ± standard deviation values of the biomarkers CA 15-3 (IU/mL), at different time points (D0, D1, and D2), Ki-67 (%), and COX-2 score among clinical stages (B, I, II, III, and IV) in CMTs. Different uppercase letters within the same column indicate statistically significant differences between groups. Different lowercase letters within the same row indicate statistically significant differences over time.

## Data Availability

The data presented in this study are available on reasonable request from the corresponding author. The data are not publicly available due to ethical and privacy restrictions related to client-owned animals and owner information.

## Abbreviations

The following abbreviations are used in this manuscript:

CA 15-3	Cancer Antigen 15-3
CMTs	Canine Mammary Tumors
COX-2	Cyclooxygenase-2
D0	Day of Mastectomy
D1	21 Days Post-Mastectomy
D2	90 Days Post-Mastectomy
H&E	Hematoxylin and Eosin
IHC	Immunohistochemistry
MUC1	Mucin 1
PCA	Principal Component Analysis
ROC	Receiver Operating Characteristic
TNM	Tumor, Node, Metastasis
WHO	World Health Organization
